# Construction of active-inert core–shell structured nanocrystals for broad range multicolor upconversion luminescence

**DOI:** 10.1038/s41598-024-57523-y

**Published:** 2024-03-26

**Authors:** Mengyao Zhu, Zhenhua Li, Xuecheng Li, Xueru Zhang, Yuxiao Wang, Haoyue Hao, Liang Li

**Affiliations:** 1 School of Physics and Optoelectronic Engineering, Shandong University of Technology, Zi Bo, 255000 People’s Republic of China; 2 Department of Physics, Harbin Institute of Technology, Harbin, 150001 People’s Republic of China

**Keywords:** Up-conversion, Core–shell structure, Emission color tuning, Energy transfer, Materials science, Optics and photonics

## Abstract

Rare earth doped up-conversion luminescent nano-materials exhibit abundant emission colors under suitable excitation condition. In this work, NaYF_4_:Er/Ho@NaYF_4_ and NaYbF_4_:Tm@NaYF_4_ nanoparticles were synthesized by co-precipitation method. The pure red emission can be realized by the designed NaYF_4_:Er/Ho@NaYF_4_ nanocrystals and the R/Gs reach 23.3 and 25 under excitations of 980 and 1550 nm lasers, respectively. The R/G declines as the power increasing with the emission color changing from red to yellow, which is due to the quick saturation of the energy levels, radiating red emissions. Meanwhile, the emission intensity of NaYbF_4_:Tm@NaYF_4_ nanocrystals increases by 58.3 folds after encasing the inert shell NaYF_4_ and the CIE color coordinate reaches (0.1646, 0.0602) under 980 nm laser excitation. Furthermore, broad range multicolor from blue to red and yellow up-conversion emissions is achieved by mixing NaYF_4_:Er/Ho@NaYF_4_ and NaYbF_4_:Tm@NaYF_4_ nanocrystals, which could be applied to colorful displaying, security anti-counterfeiting and information coding.

## Introduction

Rare earth ions doped up-conversion luminescent nano-materials, benefiting from the unique 4f energy level of trivalent rare earth ions^1^, have draw extensive attention in many research fields, like bio-imaging^2,3^, solid-state lasers^4,5^, optical temperature sensing^6,7^ and anti-counterfeiting^8,9^. Due to the remarkable physical and chemical stability, large anti-Stokes shift, multiple emission span and long emission lifetime, the rare earth ions doped materials show a broad application prospects in colorful displaying^10–20^. In up-conversion luminescent nano-materials, Er^3+^ (^2^H_11/2_/^4^S_3/2_ → ^4^I_15/2_; ^4^F_9/2_ → ^4^I_15/2_) and Ho^3+^ (^5^F_4_/^5^S_2_ → ^5^I_8_; ^5^F_5_ → ^5^I_8_) are used to achieve green and red emission colors under infrared light excitation. Tm^3+^ (^1^G_4_ → ^3^H_6_; ^1^G_4_ → ^3^F_4_) is used to obtain the blue and red emission colors^10,11^. While, the up-conversion emission colors, like green and red emissions radiated from Er^3+^, are accompanied with each other. The single emission from up-conversion phosphor is rare. Therefore, it is important and necessary to obtain a pure emission color, especially red emission color, for colorful displaying. Generally, the pure emission color can be tuned through by selecting proper ratio of co-doping rare ions and engineering local structure^13–17^. Guo et al. reported that the pure green, red and blue emissions are obtained in Yb^3+^/Ln^3+^ (Ln = Er, Ho, Tm) co-doped Gd_2_O_3_ up-conversion phosphors by adjusting the doping concentration of Er^3+^, Ho^3+^ and Tm^3+^, which is attributed to the strengthened cross relaxation processes^14^. To obtain broader range emission colors, the core–shell structured nano-materials are rational design in the past few years^11,18–22^. Jang et al. reported that the emission colors from heavily doped NaErF_4_:Tm-based core@ multi-shell nano-materials were fine tuned through changing the excitation laser from 980 to 808 and 1550 nm and the full-color emissions, including green, red and blue, were achieved via combination effects of elemental migration and photon blocking^19^. These researches indicate that the wide range emission color could be possibly achieved via rational designing core–shell structured nano-materials and tailoring the up-conversion processes. What’s more, other methods, like combining localized surface plasmon resonance (LSPR) and modifying quantum dots or dyes were also utilized to tune the emission color^23–26^.

In this work, the pure red and blue emissions are realized trough the simple double-layer structured NaYF_4_:Er/Ho@NaYF_4_ and NaYbF_4_:Tm@NaYF_4_ nanocrystals under excitation of 980 nm laser and the color can also be fine tuned from blue to red via tuning the mass ratio of the two samples, with the corresponding CIE chromatic coordinates changing from (0.1599, 0.0388) to (0.7010, 0.2813). The red to green ratios (R/Gs) reach 23.3 and 25 of NaYF_4_:Er/Ho@NaYF_4_ nanocrystals under excitation of 980 and 1550 nm lasers, respectively, which is originated to the energy transfer processes between Er^3+^ and Ho^3+^.

## Experimental

### Synthesis of NaYF_4_:Er/Ho@NaYF_4_ and NaYbF_4_:Tm@NaYF_4_ core–shell structured nanocrystals

#### Synthesis of NaYF_4_:Er/Ho nanocrystals

NaYF_4_:Er/Ho up-conversion nanocrystals were prepared through co-precipitation of the lanthanide chloride with oleic acid and 1-octadecene^27^, where YCl_3_·6H_2_O (99.9%), ErCl_3_·6H_2_O (99.9%) and HoCl_3_·6H_2_O (99.9%) were used as original materials. 1 mmol LnCl_3_·6H_2_O (Ln = 86.3%Y, 13.5% Er, 0.2% Ho), 6 ml of oleic acid and 15 ml of 1-octadecene were added into a 50 mL three-necked flask simultaneously. Heated the mixture to 150 ℃ and kept it at this temperature for 40 min. After cooling to 50 ℃, a methanol mixture of 2.5 mmol NaOH and 4 mmol NH_4_F was added to the three-necked flask and kept the reaction at this temperature for 40 min. Subsequently, the mixture was heated to 120 ℃ for 20 min to eliminate remaining water and methanol. Finally, the temperature of the mixture was increased to 310 ℃ for 1 h. The obtained nanocrystals were dispersed in 10 ml cyclohexane as the precursor solution of core–shell structure after washing with cyclohexane and ethanol in a ratio of 1:3.

#### Synthesis of NaYF_4_:Er/Ho@NaYF_4_ nanocrystals

NaYF_4_:Er/Ho@NaYF_4_ nanocrystals were prepared through the similar procedure. 1 mmol YCl_3_·6H_2_O were used as original materials. The methanol mixture of 2.5 mmol NaOH, 4 mmol NH_4_F and the precursor solution (NaYF_4_:Er/Ho) were added to the three-necked flask simultaneously. The obtained nanocrystals were washed and dried at 60 ℃ in air for 12 h for up-conversion luminescence tested.

#### Synthesis of NaYbF_4_:Tm and NaYbF_4_:Tm@NaYF_4_ nanocrystals

NaYbF_4_:0.5Tm and NaYbF_4_:0.5Tm@NaYF_4_ nanocrystals were prepared through the above procedure. Only the rare earth ions and the doped ratio differed from the previous samples.

### Measurements and characterization

The X-ray powder diffraction (XRD) patterns were recorded using a Bruker D8 diffractometer to investigate the phase purity and phase structure of the samples. The transmission electron microscope (TEM) images were recorded by a Talos F200X G2 field emission electron microscope to investigate the morphologies of the samples. The 980 nm laser (EC31439), using to excite the sample, was purchased from Changchun New Industries Optoelectronics Tech Co., Ltd. The 1550 nm laser (BTW DS2-21312110), using to excite the sample, was purchased from Beijing Kipling Photoelectric technology Co., Ltd. The up-conversion emission spectra of the samples were measured through the fiber optic spectrometer purchased from Chen Xu instrument Co., Ltd (Type: ST4000). The time-dependent emission profiles of the samples were recorded using iHR550 grating spectrometer with a DSO5032A Digital Storage Oscilloscope.

## Results and discussion

### Structure and morphological characterization

**Figure 1:**
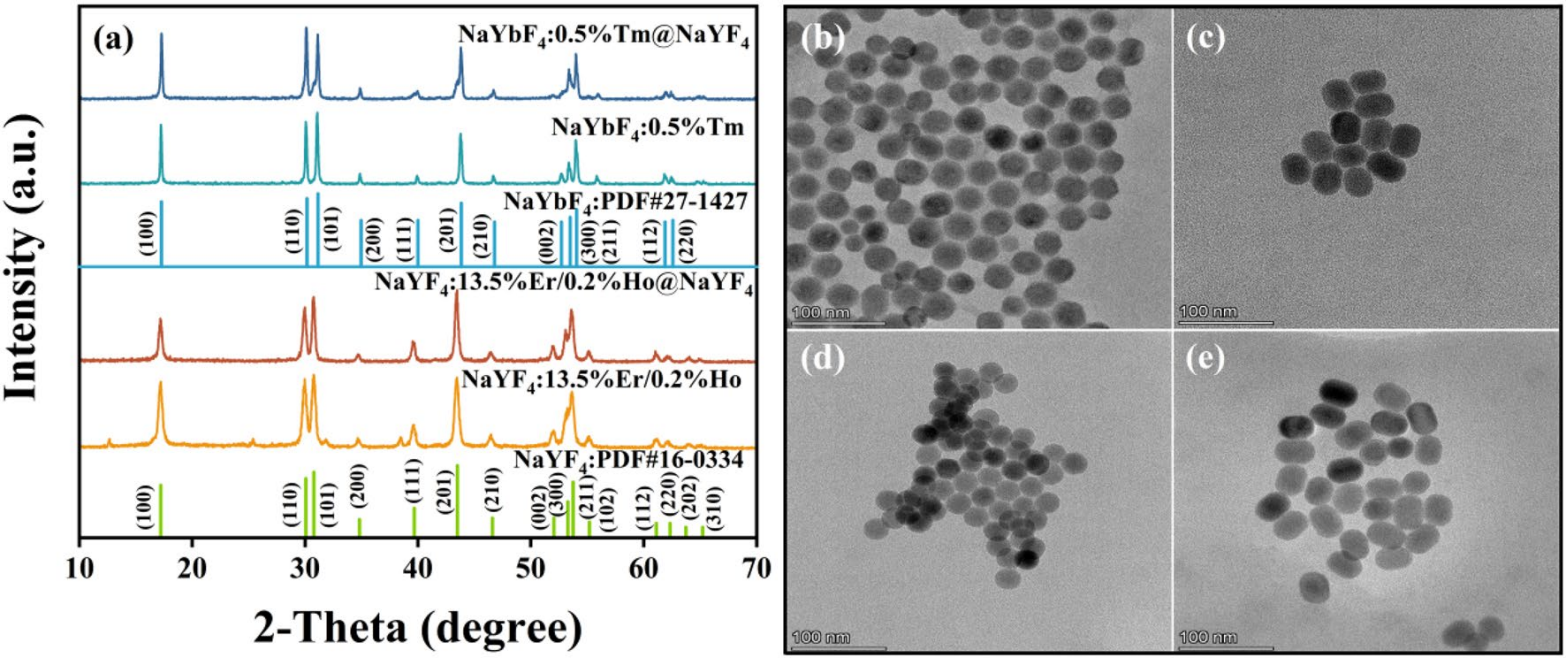
(**a**) XRD patterns NaYF_4_:Er/Ho, NaYF_4_:Er/Ho@NaYF_4,_ NaYbF_4_:Tm and NaYbF_4_:Tm@NaYF_4_; TEM mages of (**b**) NaYbF_4_:Tm (**c**) NaYbF_4_:Tm@NaYF_4_ (**d**) NaYF_4_:Er/Ho (**e**) NaYF_4_:Er/Ho@NaYF_4_ nanocrystals.

XRD patterns of all samples are illustrated in Fig. 1a. Compared with the two kinds of standard hexagonal phase NaYF_4_ (JCPDS No.16–0334) and NaYbF_4_ (JCPDS No.27–1427), NaYF_4_:Er/Ho and NaYF_4_:Er/Ho@NaYF_4_ nanocrystals are pure hexagonal phase NaYF_4_. NaYbF_4_:Tm and NaYbF_4_:Tm@NaYF_4_ nanocrystals are pure hexagonal phase NaYbF_4_. Figure 1b–e show TEM images of NaYbF_4_:Tm, NaYbF_4_:Tm@NaYF_4_, NaYF_4_:Er/Ho and NaYF_4_:Er/Ho@NaYF_4_ nanocrystals, respectively. As shown in Fig. 1b, the obtained NaYbF_4_:Tm nanocrystals is composed of monodisperse sphere and the average diameter is ~ 28.5 nm. After coating by inert shell NaYF_4_, the NaYbF_4_:Tm@NaYF_4_ nanocrystals are prepared, where the nanospheres become ellipsoid and the average size increases to ~ 41.1 × 31.9 nm. As revealed in Fig. 1d, the average size of single core NaYF_4_:Er/Ho nanocrystals is ~ 26.1 nm and the nanocrystals distribute homogeneously. The morphology of NaYF_4_:Er/Ho@NaYF_4_ nanocrystals is similar to NaYbF_4_:Tm@NaYF_4_ nanocrystals and the average size is ~ 39.9 × 30.3 nm. The size distribution diagrams of these nanocrystals are shown in Fig. S1.

### Up-conversion luminescent properties

#### Up-conversion emission spectra and emission color of NaYF_4_:Er/Ho, NaYF_4_:Er/Ho@NaYF_4_ nanocrystals

**Figure 2:**
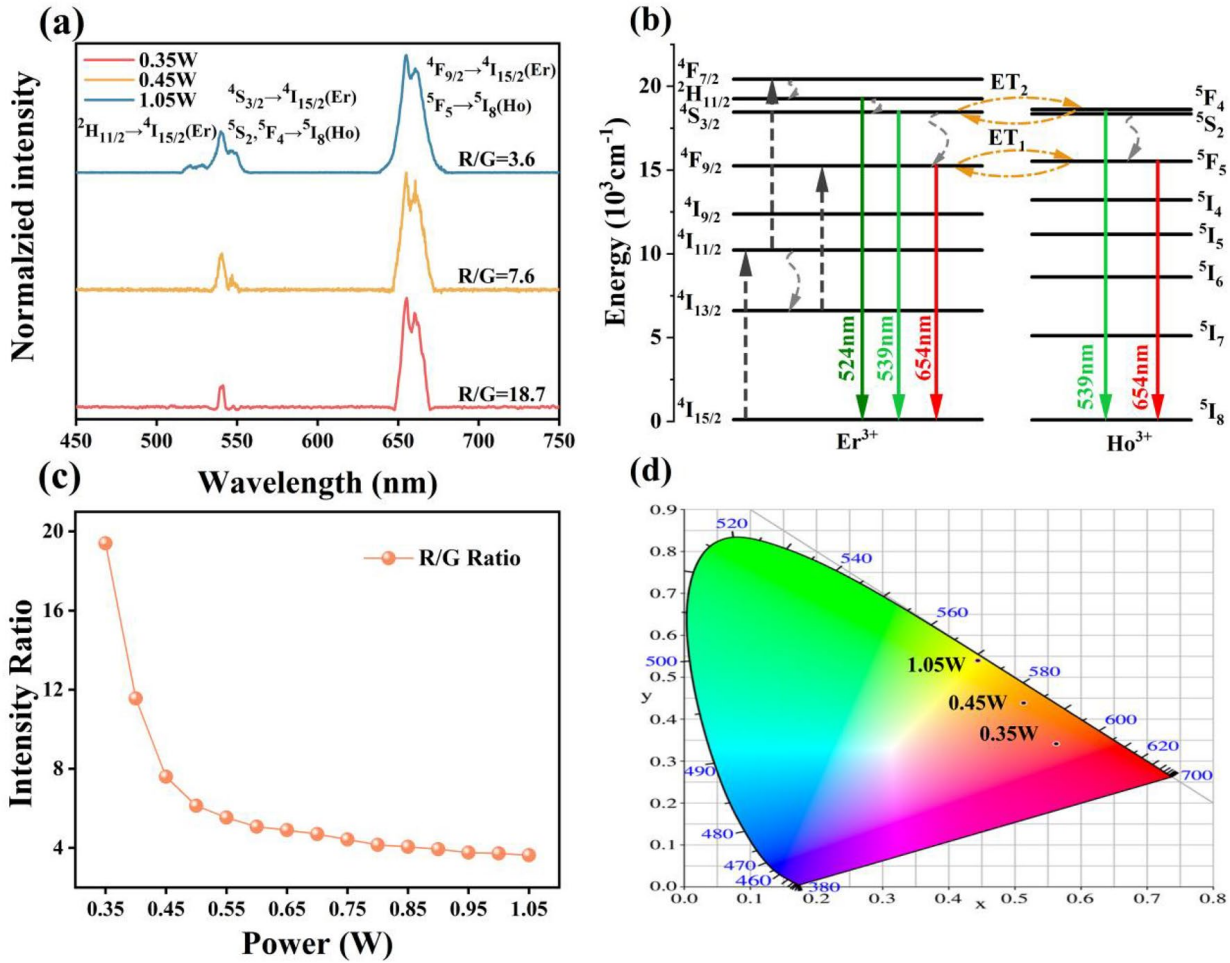
(**a**) Normalized emission spectra of NaYF_4_:Er/Ho nanocrystals under 980 nm laser excitation; (**b**) The proposed up-conversion and energy transfer processes; (**c**) R/G variation and (**d**) the corresponding CIE chromatic coordinates at different powers.

The Er^3+^ and Ho^3+^ co-doping NaYF_4_ nanocrystals are responsive to the excitation wavelengths of 980 and 1550 nm. Figure 2a shows the up-conversion emission spectra of the NaYF_4_:Er/Ho nanocrystals under 980 nm laser excitation (0.35W, 0.45W, 1.05W), where the emission spectra were normalized at 654 nm. The typical emission bands of Er^3+^ located at 524, 540 and 655 nm are observed, which corresponding to the radiated transitions of ^2^H_11/2_ → ^4^I_15/2_, ^4^S_3/2_ → ^4^I_15/2_ and ^4^F_9/2_ → ^4^I_15/2_ respectively. What more, comparing with the emission spectrum of NaYF_4_:Er (shown in Fig. S2), part of the emissions from the NaYF_4_:Er/Ho nanocrystals belong to the green-emitted ^5^F_4_, ^5^S_2_ → ^5^I_8_ transitions and the red-emitted ^5^F_4_ → ^5^I_8_ transition in Ho^3+^. The R/G ratio are 18.7, 7.6 and 3.6 as the power of 980 nm laser changes to 0.35, 0.45 and 1.05, respectively. Comparing to Er^3+^ doped NaYF_4_ nanocrystals, the value of R/G increases obviously in Er^3+^ and Ho^3+^ co-doped NaYF_4_ nanocrystals, which is due to the new energy transfer processes between Er^3+^ and Ho^3+^ ions^28^. In the Er^3+^/Ho^3+^ co-doped system, Er^3+^ ions can absorb the energy of 980 nm laser as a kind of sensitizer and transfer part of energy to the co-doped Ho^3+^. Based on the well energy level overlap between the Er^3+^ and Ho^3+^, the energy transfer ET1 (Er^3+^: ^4^F_9/2_ → Ho^3+^: ^5^F_5_), ET2 (Er^3+^: ^2^H_11/2_^/4^S_3/2_ → Ho^3+^: ^5^F_4_/ ^5^S_2_) and the ground state absorption (Er^3+^: ^4^I_15/2_ → ^4^I_11/2_), excited state absorption (Er^3+^: ^4^I_11/2_ → ^2^H_11/2_^/4^S_3/2_ and ^4^I_13/2_ → ^4^F_9/2_) are shown in Fig. 2b. As shown in Fig. 2c, the R/G decreases from 18.7 to 3.6 with the rise of power. The reason for the decrease could be explanation as follows. The R/G value mainly depends on the depletion of the energy level ^4^I_11/2_ (Er^3+^). There are two channels for the depletion of ^4^I_11/2_, including excited state absorption ^4^I_11/2_ → ^4^F_7/2_ and non-radiative relaxation ^4^I_11/2_ → I_13/2_. When the pump power is very small, the up-conversion process of ^4^I_11/2_ mainly contributes to the population of the energy level I_13/2_ due to the non-radiative relaxation and then populate the red light-emitting level through excited state absorption I_13/2_ → ^4^F_9/2_. With the increase of pumping power, a considerable part of the electrons on ^4^I_11/2_ will populate the green light-emitting level through the up-conversion process, which in turn reduces the proportion of red light-emitting level^29,30^. As shown in Fig. 2d, the corresponding CIE chromatic coordinate changes from red to yellow as the power increases and the detail CIE chromatic coordinates are displayed in Table S1.

**Figure 3:**
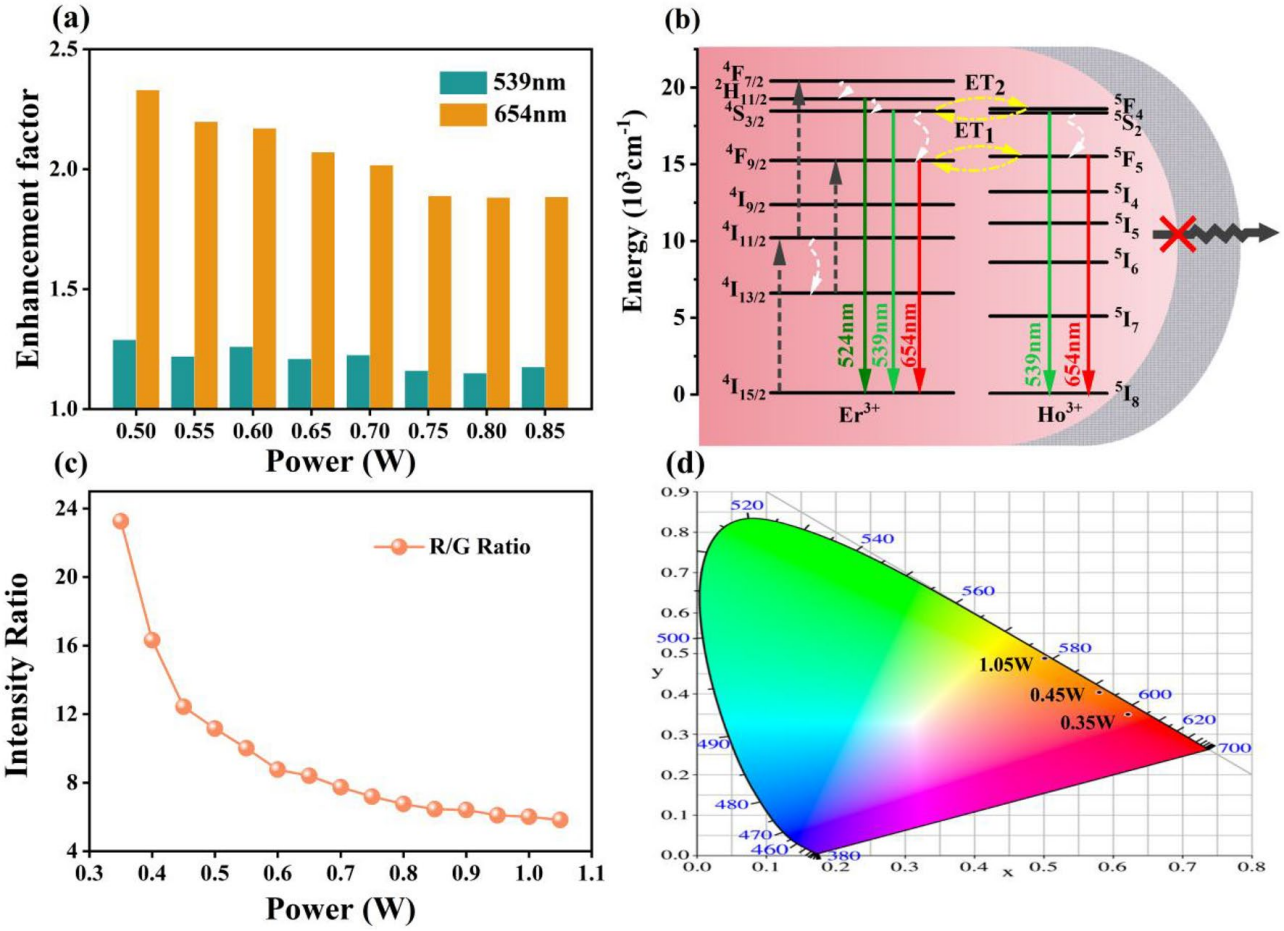
(**a**) The enhancement factor (**c**) dependence of R/G and (**d**) the corresponding CIE chromatic coordinates of NaYF_4_:Er/Ho@NaYF_4_ nanocrystals under 980 nm laser excitation with different powers; (**b**) The proposed up-conversion processes and surface quenching.

Figure 3a shows the emission intensity enhancement factor of NaYF_4_:Er/Ho@NaYF_4_ nanocrystals. The enhancement factors of green (539 nm) and red (654 nm) emissions show a slow downward trend as laser power increases. And the enhancement factor of green emissions decreases from 1.29 to 1.18. The enhancement factor of red emission decreases from 2.33 to 1.88. The enhancement factor of the red declines faster with increasing power than that of the green emissions. The emission enhancement is due to the suppression of surface quenching, as shown in Fig. 3b. The emission lifetimes of green and red emissions change longer as the NaYF_4_ shell are coated (as shown in Fig. S3), which confirms the decline of surface quenching^31^. And the more obvious enhancement of red emission might be originated to the increased ET1 process. It is worth to mention that the R/G decreases from 23.3 to 5.8 with the increasing of power from 0.35 to 1.05 W (as shown in Fig. 3c). As a result, comparing to NaYF_4_:Er/Ho nanocrystals, the corresponding CIE chromatic coordinate changes to deep red region (as shown in Fig. 3d and Table S2).

**Figure 4:**
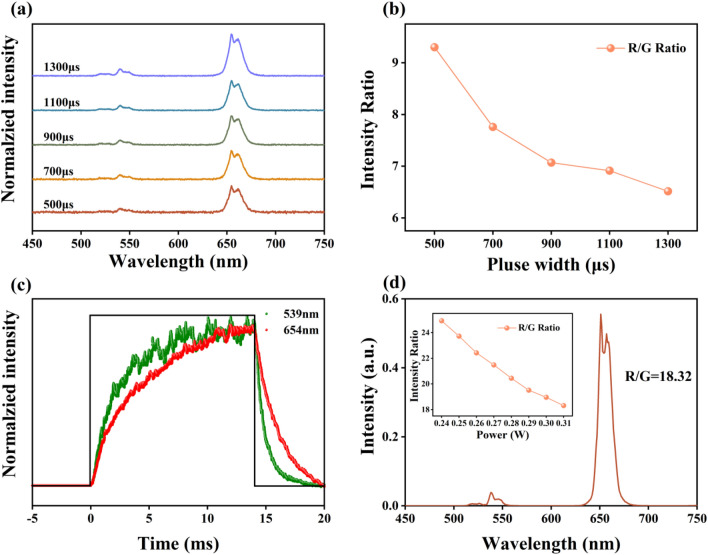
(**a**) Normalized emission spectra and (**b**) the R/G of NaYF_4_:Er/Ho@NaYF_4_ nanocrystals under 980 nm laser excitation at different pulse width; (**c**) Time-dependent green and red emission profiles of NaYF_4_:Er/Ho@NaYF_4_ nanocrystals; (**d**) The up-conversion emission spectra of NaYF_4_:Er/Ho@NaYF_4_ nanocrystals under 1550 nm laser excitation, the insert show the R/G with different excitation powers.

To investigate the non-steady up-conversion processes, the up-conversion emission spectra of NaYF_4_:Er/Ho@NaYF_4_ at different pulse widths from 500 to 1300 μs were tested (the pulse frequency was fixed at 600 Hz). As shown in Fig. 4a, the green emissions are weak and become obviously as the pulse width enlarges. And the value of R/G ratio decreases from 9.3 to 6.5 with the pulse duration times increasing from 500 to 1300 μs, as shown in Fig. 4b. This phenomenon is different to the tendency of other rare ions doped materials, like Ho(Er)/Yb, where the R/G rises as the pulse widths increase^32,33^. To explain the reason why the R/G declines with pulse width rise, we investigated the non-steady state behavior of the sample under 980 nm laser excitation. As shown in Fig. 4c, the intensity of green and red emissions rise slowly under excitation. And the rise time is longer than the nanocrystals without NaYF_4_ shell (as shown in Fig. S4), indicating that the NaYF_4_ shell intensifies the ET1 and ET2. The similar rise tendency of green and red emissions, unlike the shorter rise time of green emissions in other reports^32–34^, make the different R/G change tendency with pulse width increasing. The reason of the slower rise time of this sample is that the ET1_,_ ET2, back-ET1 (BET1)_,_ back-ET2 (BET1) and non-radiative relaxation processes, as shown in Fig. 3b, repopulate the energy levels of ^4^F_9/2_, ^2^H_11/2_, ^4^S_3/2_, ^5^F_5_, ^5^F_4_/ ^5^S_2_.

Upon changing the excitation wavelength to 1550 nm, the emission spectra of NaYF_4_:Er/Ho@NaYF_4_ nanocrystals are detected under 1550 nm laser. As shown in Fig. 4d, the value of R/G reaches 25 under low power excitation and show the similarity decrease tendency as laser power increases. Comparing with sample under 980 nm laser excitation, the larger value of R/G is obtained under 1550 nm laser excitation. This phenomenon can be interpreted with the original populations of energy levels of Er^3+^: ^4^F_9/2_, ^2^H_11/2_, ^4^S_3/2_, which can be deduced from the up-conversion emission spectrum of NaYF_4_:Er under 1550 nm laser excitation (as shown in Fig. S5). The large R/G value indicates that the NaYF_4_:Er/Ho@NaYF_4_ nanocrystals can be used as red phosphors under 980 and 1550 nm laser excitation.

#### Up-conversion emission spectra and emission color of NaYbF_4_:Tm and NaYbF_4_:Tm@NaYF_4_ nanocrystals

**Figure 5:**
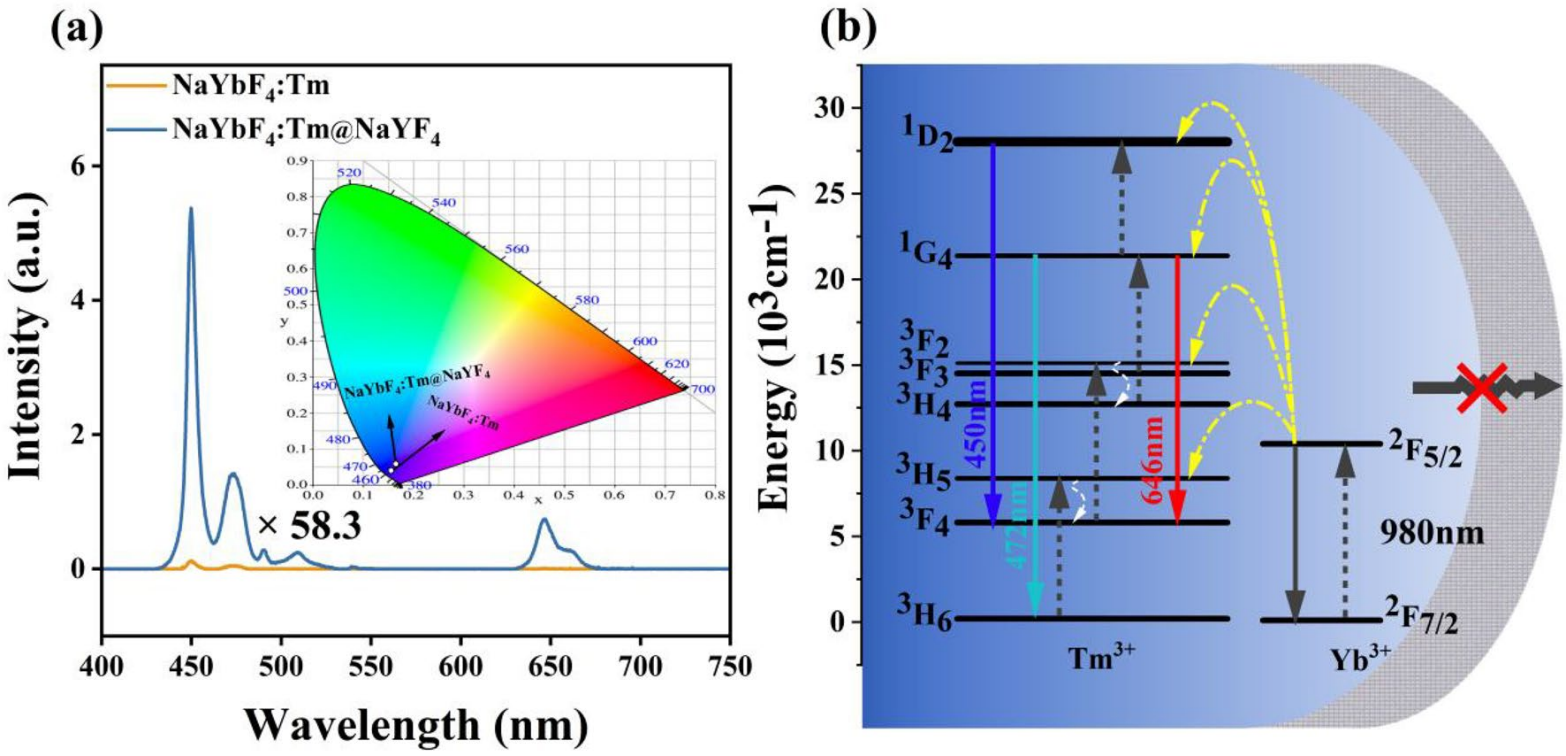
(**a**) The up-conversion emission spectra of NaYbF_4_:Tm and NaYbF_4_:Tm@NaYF_4_ nanocrystals, the insert show the corresponding CIE chromatic coordinates; (**b**) The proposed up-conversion luminescent mechanism.

To obtain the pure blue phosphors, the bare core NaYbF_4_:Tm and core–shell NaYbF_4_:Tm@NaYF_4_ structured nanocrystals were prepared. It can be observed in Fig. 5a, after coating the inert shell with NaYbF_4_:Tm, the emission intensity of NaYbF_4_:Tm@NaYF_4_ increases by 58.3 fold. It should be mentioned that the NaYF_4_ shell plays an important role in inhibiting surface quenching and increasing emission intensity. The intense blue emissions at 450 and 472 nm makes the emission color display pure blue, as shown in the insert of Fig. 5a (the detail CIE chromatic coordinates are displayed in Table S3). The relevant up-conversion processes are displayed in Fig. 5b. The efficient energy transferred from Yb^3+^ can be used to populated the energy levels of ^1^D_2_, ^1^G_4_ and then radiated intense blue emissions. What’s more, the emission color almost unchanges with the increasing of laser power, as shown in Fig. S6, which provides the possibility for the mixed materials to regulate emission color.

#### Broad bange upconversion emission spectra and emission color

**Figure 6:**
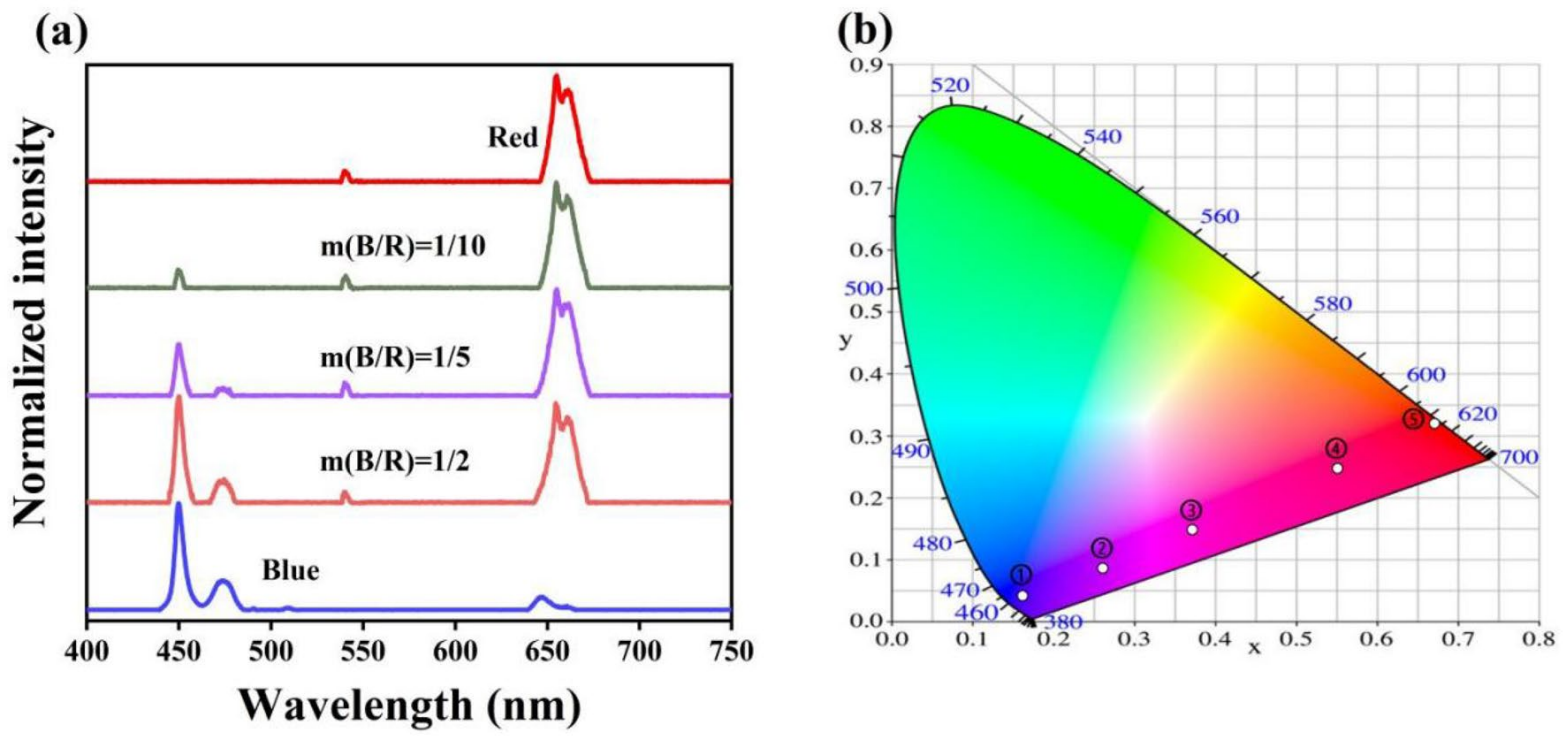
(**a**) Normalized emission spectra (**b**) the corresponding CIE chromatic coordinates of samples with fixed mass ratio under 980 nm laser excitation.

In order to realize the broad domain multicolor up-conversion luminescence, these two types of distinct phosphors, NaYbF_4_:Tm@NaYF_4_ and NaYF_4_:Er/Ho@NaYF_4_ nanocrystals, were dissolve in alcohol and grind in accordance with fixed mass ratio: ① only NaYbF_4_:Tm@NaYF_4_ nanocrystals, ② 1:10, ③ 1:5, ④ 1:2 and ⑤ only NaYF_4_:Er/Ho@NaYF_4_ nanocrystals. The emission spectra of the mixed samples are shown in the Fig. 6a, and the corresponding CIE chromatic coordinates are presented in Fig. 6b. As expected, Fig. 6b shows a wide range of color diversity from blue to red, including blue (0.1613, 0.0421), purple (0.2604, 0.0872), magenta (0.3722, 0.1489), crimson (0.5506, 0.2474) and red (0.6700, 0.3202). We also investigated the luminescence properties of these samples under excitation with different 980 nm laser power, as shown in Fig. S7. As the power increases, the CIE chromatic coordinates go to the red and green region (the detail CIE chromatic coordinates are displayed in Table S4), which eventually occupy over one-third of the entire chromaticity diagram. This result indicates that the composites may find applications in colorful displaying and anti-counterfeiting.

## Conclusions

In summary, we designed double-layer core–shell structure to investigate the effect of excitation condition on up-conversion emission spectra and emission color. The pure red emission can be realized by the designed NaYF_4_:Er/Ho@NaYF_4_ nanocrystals under 980 or 1550 nm laser excitation. And the R/G declines as the power of 980 nm laser increases, with the emission color changing from red to yellow, which can be interpreted by the quick saturation of the energy levels, radiating red emissions. Because of the ET, BET and non-radiative relaxation processes among Ho^3+^ and Er^3+^, the R/G also decreases as the pulse width rises. Meanwhile, the up-conversion luminescence of NaYbF_4_:Tm@NaYF_4_ phosphors under 980 nm laser excitation were also studied. After encasing the inert shell NaYF_4_, the emission intensity from NaYbF_4_:Tm@NaYF_4_ nanocrystals increases by 58.3 folds. A wide range emission colors from blue to red, including blue, purple, magenta, crimson and red are realized through tuning the mass ratio of two samples. As the power increasing, the CIE chromatic coordinates go to the red and green region and eventually occupy over one-third of the entire chromaticity diagram. These results indicate the potential applications of these materials in various fields, including colorful displaying, security anti-counterfeiting and information coding.

### Supplementary Information

Supplementary Information.

## Data Availability

The datasets used and/or analysed during the current study available from the corresponding author on reasonable request.
