# Supplementary material for: Construction of active-inert core–shell structured nanocrystals for broad range multicolor upconversion luminescence
Source: Sci Rep. 2024 Mar 26;14:7099. doi: 10.1038/s41598-024-57523-y (PMC10966044; doi:10.1038/s41598-024-57523-y)
Supplement: Supplementary file 1 — Supplementary Information. [file 41598_2024_57523_MOESM1_ESM.docx]

**Supporting Information**

# Construction of Active-inert Core-shell Structured Nanocrystals for Broad Range Multicolor Upconversion Luminescence

Mengyao Zhu,^a^ Zhenhua Li,^b^ Xuecheng Li,^a^ Xueru Zhang,^b^ Yuxiao Wang,^b^ Haoyue Hao,^a^*^1^ Liang Li^a^*^2^

^a^ *School of Physics and Optoelectronic Engineering,* *Shandong University of Technology, Zi Bo 255000, PR China*

*^b^ Department of Physics,* *Harbin Institute of Technology, Harbin 150001, PR China*

* *Corresponding author*

^1^ [haohao_yue@163.com](mailto:powerhit1990@163.com)

^2^ [liangli@sdut.edu.cn](mailto:liangli@sdut.edu.cn)


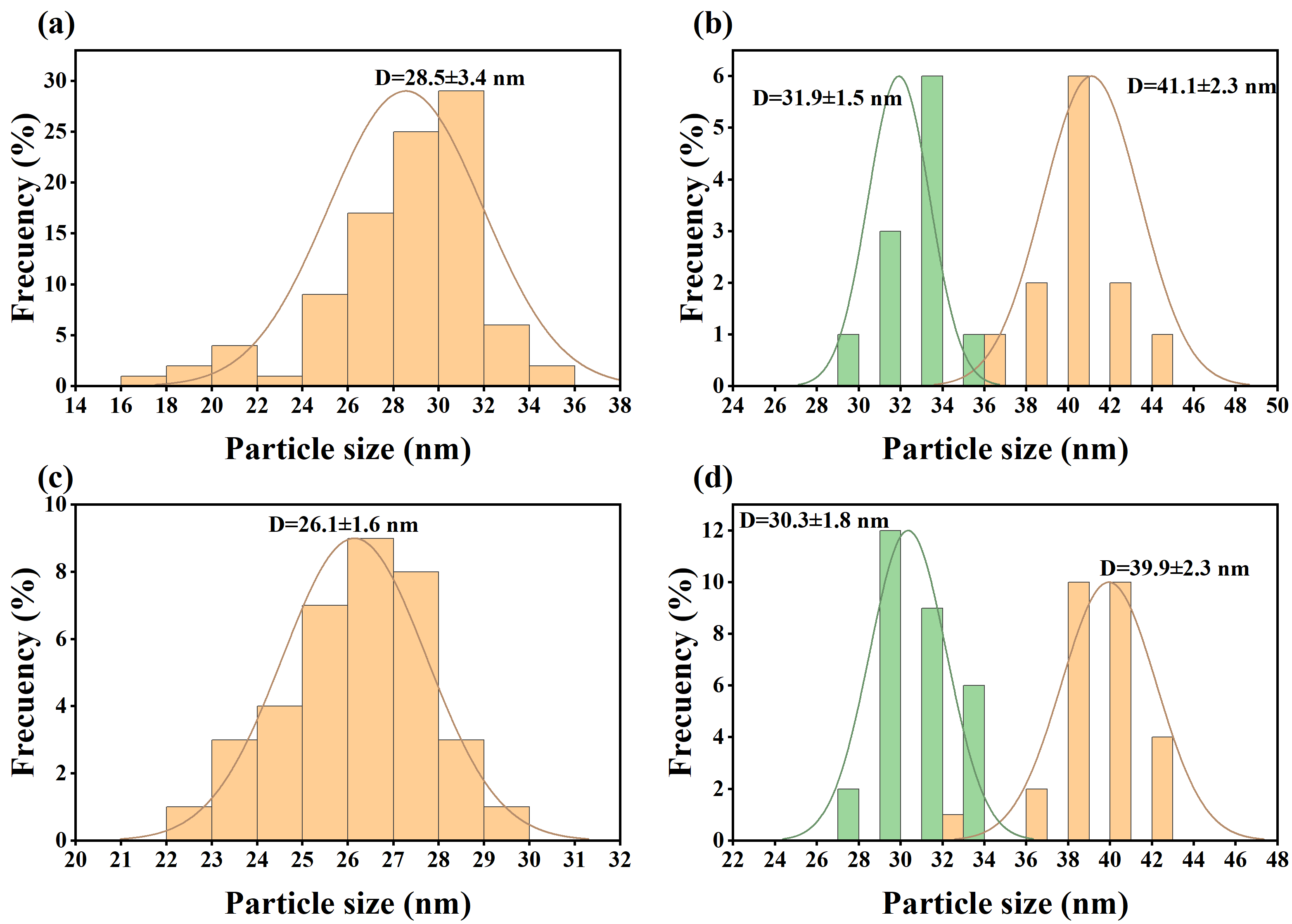


**Fig. S1.** The size distribution diagrams of (a)NaYbF_4_:Tm (b) NaYbF_4_:Tm@NaYF_4_ (c) NaYF_4_:Er/Ho (d) NaYF_4_:Er/Ho@NaYF_4_ nanocrystals.


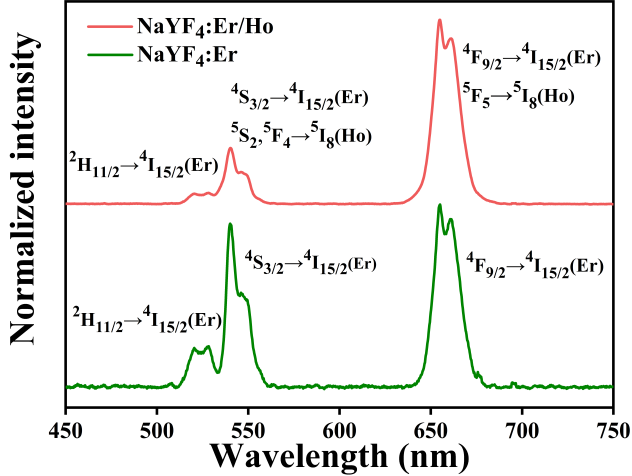


**Fig. S2.** Normalized emission spectra of NaYF_4_:Er/Ho and NaYF_4_:Er under 980nm laser excitation.

**
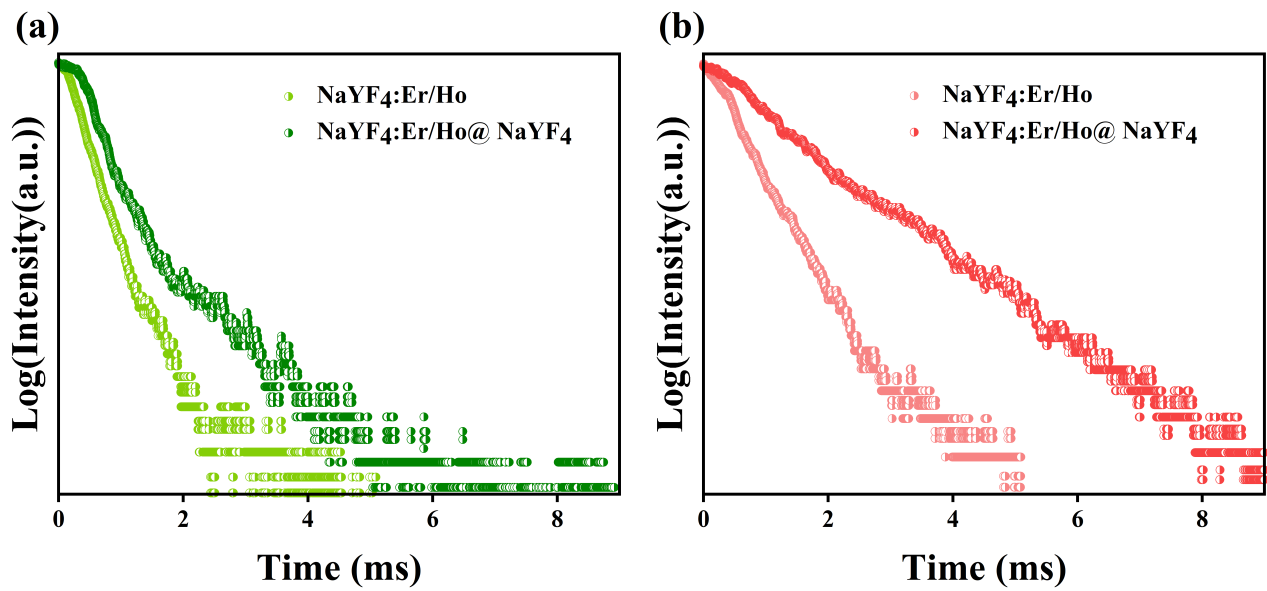
Fig.S3.** The emission lifetimes at (a) 539 nm and (b) 654 nm of NaYF_4_:Er/Ho and NaYF_4_:Er/Ho@NaYF_4_ nanocrystals.


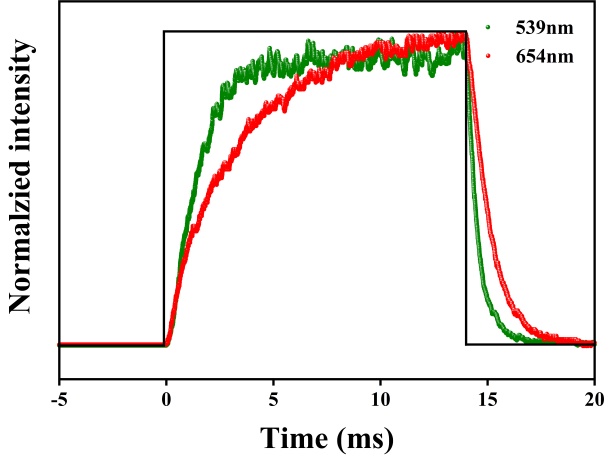


Fig.S4. Time-dependent emission profiles of NaYF_4_:Er/Ho nanocrystals.


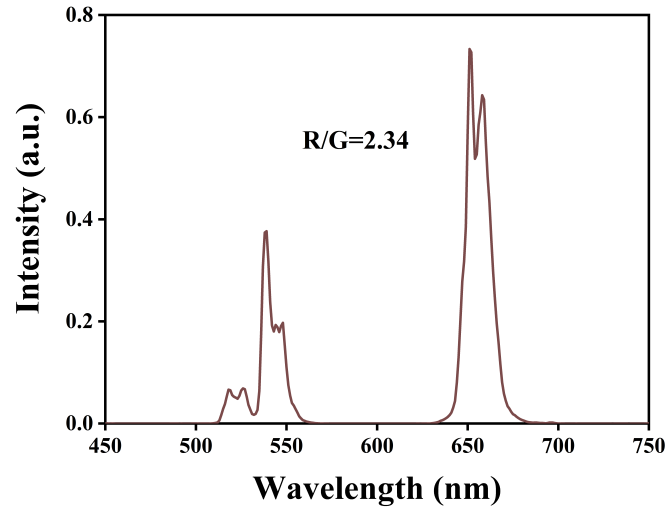


**Fig. S5.** The up-conversion emission spectrum of NaYF_4_:Er under 1550 nm laser excitation.


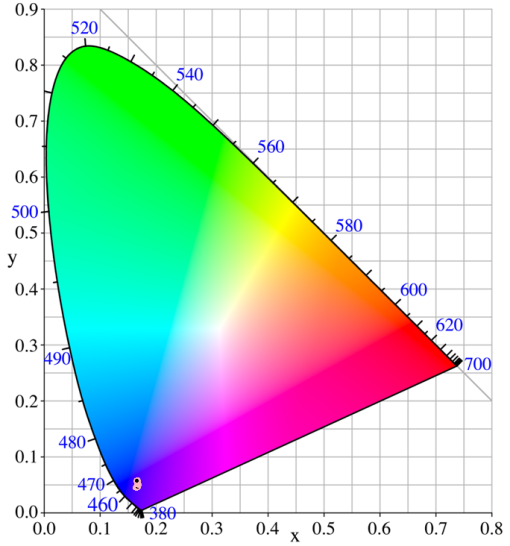


**Fig.S6.** CIE coordinates of NaYbF_4_:Tm@NaYF_4_ nanocrystals under 980 nm laser excitation.


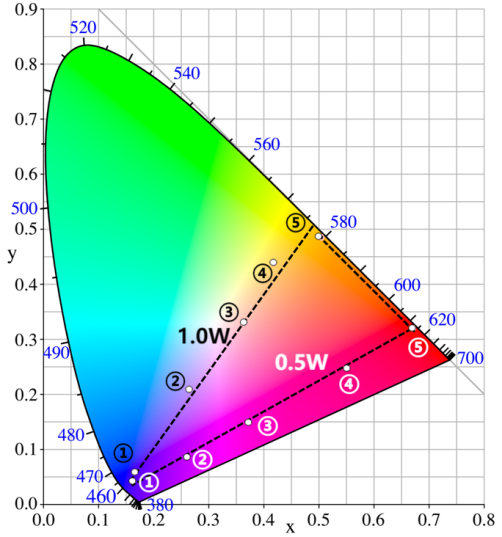


**Fig.S7.** CIE coordinates of samples under different power of 980 nm laser excitation.

**Table S1.** CIE color coordinates of NaYF_4_:Er/Ho under different excitation powers.

| Power of 980 nm  laser (W) | Chromaticity  coordinates (x, y) |
| --- | --- |
| 0.35 | (0.5631, 0.3413) |
| 0.45 | (0.5130, 0.4392) |
| 1.05 | (0.4440, 0.5402) |

**Table S2.** CIE color coordinates of NaYF_4_:Er/Ho@NaYF_4_ under different excitation powers.

| Power of 980 nm  laser (W) | Chromaticity  coordinates (x, y) |
| --- | --- |
| 0.35 | (0.6213, 0.3498) |
| 0.45 | (0.5806, 0.4044) |
| 1.05 | (0.5017, 0.4877) |

**Table S3.** CIE color coordinates of NaYbF_4_:Tm and NaYbF_4_:Tm@NaYF_4_ under 980 nm laser excitation at 1.10 W.

| Nanocrystals | Chromaticity  coordinates (x, y) |
| --- | --- |
| NaYbF_4_:Tm | (0.1542, 0.0409) |
| NaYbF_4_:Tm@NaYF_4_ | (0.1646, 0.0602) |

**Table S4.** CIE color coordinates of all samples under different excitation powers.

| Power of 980 nm  laser (W) | Chromaticity  coordinates (x_1_, y_1_) | Chromaticity  coordinates (x_2_, y_2_) | Chromaticity  coordinates (x_3_, y_3_) | Chromaticity  coordinates (x_4_, y_4_) | Chromaticity  coordinates (x_5_, y_5_) |
| --- | --- | --- | --- | --- | --- |
| 0.50 | (0.1613, 0.0421) | (0.2604, 0.0872) | (0.3722, 0.1489) | (0.5506, 0.2474) | (0.6700, 0.3202) |
| 1.00 | (0.1652, 0.0581) | (0.2641, 0.2095) | (0.3650, 0.3293) | (0.4172, 0.4382) | (0.5046, 0.4856) |
